# Mapping internet literacy skills of digital natives: A developing country perspective

**DOI:** 10.1371/journal.pone.0249495

**Published:** 2021-04-20

**Authors:** Saira Hanif Soroya, Abdus Sattar Ahmad, Shakil Ahmad, Muhammad Shahid Soroya

**Affiliations:** 1 Institute of Information Management, University of the Punjab, Lahore, Pakistan; 2 Minhaj University Lahore, Lahore, Pakistan; 3 Prince Sultan University, Riyadh, Saudi Arabia; 4 Minhaj University Lahore, Lahore, Pakistan; National Textile University, PAKISTAN

## Abstract

Over the last three decades, Information and Communication Technologies (ICTs) have changed the world in all walks of life. It has not only influenced the ways of human communication but also changed the way of learning. However, to utilize the facility of the Internet in effective manners, people need a certain set of skills called “Internet Literacy Skills”. The purpose of the study was to explore the level of Internet literacy skills of Undergraduate first-year students (Digital Natives) of the University of the Punjab, Lahore, Pakistan. The study is quantitative, and data were gathered through questionnaires. A total of 180 students from three disciplines i.e. Pure Sciences, Social Sciences, and Arts and Humanities were approached for the final data collection. Descriptive (mean and standard deviation) and inferential statistics (regression analysis) were applied to analyze the collected data. Results further revealed that respondents possess very good knowledge to identify legal and illegal activities and information on the Internet. Findings of the study, not only reported the Internet literacy skills of digital natives but also helped to come up with a theoretical model that may be useful to design an efficient and effective Internet literacy module/subject to help students increase their Internet use-related skills.

## Introduction

Over the last three decades, computer, information, and communication technologies (ICTs) have changed the world in all walks of life. It has not only influenced the ways of human communication but also changed the way of learning. In the beginning, the Internet was available in very limited areas and to a very limited number of people. The cost to access the Internet was high. But as technology got cheaper, access to the Internet has become easier and affordable. People from all over the world can access the Internet easily and at a low cost. The Internet provides fast access to information on all subject areas from a remote location with just one click. People use the Internet for communication or to share information through social media networks like Facebook, Twitter; and through email accounts provided by different hosts like Google and Yahoo mail service. They use the Internet to search for information related to their studies, for instance searching an article on google scholar, and to get information about the current trends of clothes and shoes, entertainment, leisure, and health. Moreover, the Internet provides the facility to store a lot of information on the virtual space and users can access their information anytime and anywhere in the world.

The Internet is mostly used by young people, and particularly students who are more inclined to use Internet resources for education, social interaction, and entertainment [1]. Prensky [2] opined that young students have more exposure to information technology (IT) and the Internet as compared to previous generations, therefore, they are known as digital natives, as they are grown up in the digital age. Digital natives are heavy users of computers and the Internet through various devices i.e. mobile, tablet, laptop, and desktop.

The use of IT and the Internet is also common in the education sector. Hussain [3] reported that research and education developments have been increased using the Internet in higher education. The Internet has made it easy to virtually share research findings and other information with others. The Internet as an essential universal source to retrieve and disseminate information helps students to broaden their educational experiences [4].

However, to perform all these actions on the Internet, users need a set of skills necessary to utilize the facility of the Internet. These skills are known as Internet literacy competence. Machin-Mastromatteo [5] suggested that there is a need to think beyond information literacy and it is important to integrate digital literacy into information literacy trainings.

Different terminologies are used by different authors for Internet literacy. Among those, digital literacy [6,7], computer literacy [8], web literacy [9], and media literacy [10] are commonly used to represent the term Internet literacy. However, all these terminologies are used as different descriptions for similar concepts that are “the skills necessary to make effective use of computer and the Internet”. These skills are inevitable for every person to survive in the current era of information and communication technologies (ICTs). Students particularly need to learn these Internet literacy skills so that they can better utilize the information available on the Internet, they can communicate easily through the Internet, and they can use the Internet for other educational activities effectively and efficiently.

Different researchers [11,12] have defined Internet literacy differently in their ways, however, in 2013 the definition provided by the Ministry of Internal Affairs and Communications Japan [13] seems more comprehensive, as the ability necessary to access the Internet while incorporating the following three points: (1) the ability to address illegal and harmful contents on the Internet appropriately, (2) the ability to communicate on the Internet appropriately, and (3) the ability to protect their privacy and perform security measures.

Globally several studies have been conducted to observe the Internet use behavior of the students in the universities [14–18]. The researchers identified that lack of computer skills for searching and retrieval of information, low knowledge about information privacy and security are common problems that students usually face. Similarly, Internet literacy has been measured in different contexts and of different age groups i.e. For Example, older adults [19], and adolescents from the United States [20]. Lately, different social groups i.e. Indonesian school teachers [21], Chinese and German students [22], Nigerian pharmacists [23] have also been a part of the investigation regarding the studies about Internet literacy skills.

In Pakistan, many institutions have invested huge amounts of money to employ the facilities of ICTs in their institutions. They have invested a considerable amount in the latest computer labs, buildings, furniture, and hiring experts so that they can provide Internet facilities to their staff and students.

University of the Punjab (PU), Lahore is also on the way to provide the students and staff with Internet facilities. There are computer labs in all departments/institutes/colleges which provide Internet facilities to the students. Besides these computer labs, there is a computer lab in the central library that is accessible by all the members of the library. These computer labs provide a wide range of access to information resources subscribed by the Higher Education Commission, Pakistan. Students use these labs for research purposes, communication, information searching, and socialization. The use of available databases and technologies requires considerable knowledge and skills and understanding about how to use them effectively. The library plays an important role in helping students to meet these skills. They conduct different workshops like how to use Endnote for reference management and how to use the library catalog for searching information resources available in the library and they teach the students about different information resources and the ways to effectively use them.

Despite the increase in the daily use of the internet among different Pakistani social groups, particularly students, no empirical investigation on the Internet literacy skills of university students at any point could be found. Although a handful of studies are available on information literacy skills of different social groups including faculty members and students. A research gap was felt, and the researchers decided to conduct a study to examine the Internet literacy skills of undergraduate students (digital natives) of the University of the Punjab (PU). This study will highlight the importance of Internet Literacy Skills, update the current status of IL skills of students, and will open a new debate through mapping a model and serving as a baseline study for future research.

### Research objectives

The research objectives of the study are:

1. To ascertain students’ ability to address the illegal and harmful contents on the internet appropriately.
2. To examine students’ ability to communicate on the Internet appropriately.
3. To assess students’ ability to protect their privacy and perform security measures.
4. To map a model predicting antecedents of Internet literacy based on the study findings.

### Research hypotheses

The first three objectives of the study were descriptive in nature and were measured through mean and standard deviation, whereas to measure the fourth research objective following research hypotheses were developed.

H1: Students with a high frequency of Internet use are more likely to possess a higher level of knowledge to determine the legality of content.
H2: Students with a high level of knowledge about the legality of the content are more able to address illegal and harmful content on the Internet appropriately.
H3: High frequency of Internet use has a positive significant impact on online communication channels’ exposure.
H4: Students with a high frequency of online communication channels exposure are significantly proficient in communication over the Internet.
H5: Students with a high frequency of Internet use are more likely to possess a higher level of awareness about data privacy.
H6: Students with a high level of awareness about data privacy are more likely to exercise information control.

**Fig 1:**
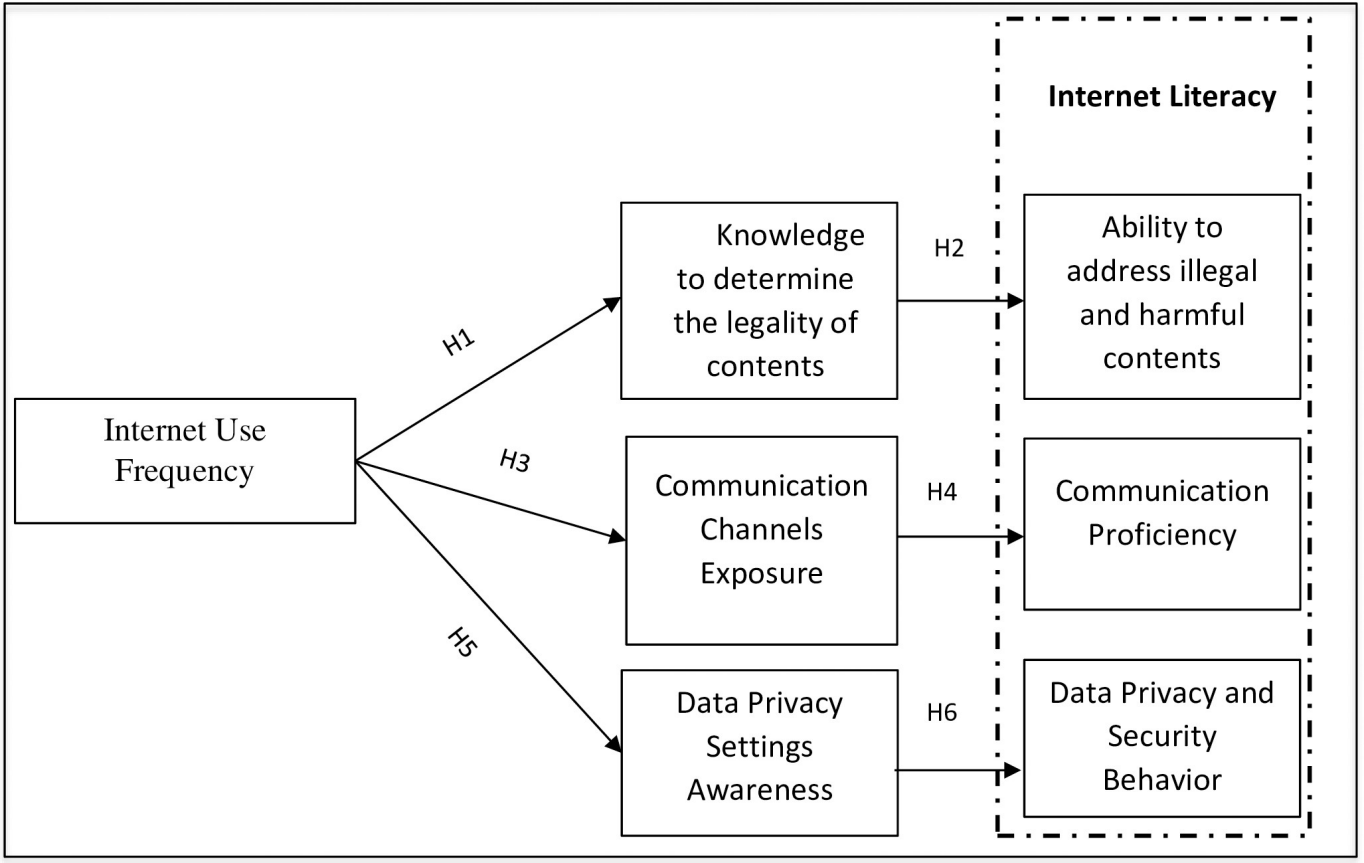
Proposed hypothetical model.

Based on the above-mentioned hypotheses, the following model was proposed (see Fig 1).

## Materials and methods

The study was quantitative and the survey research method was adopted to conduct the present study. This is a quantifiable method for gathering desired information from a group of respondents by asking several study questions and data can be collected in different forms like respondents’ opinions, beliefs, points of view, and attitudes. Furthermore, several related studies [24–28] have successfully used similar methods for such type of data collection from different social groups.

First-year students of the University of the Punjab, Lahore enrolled in the BS program were considered the population of this study. The reason to select BS first-year students through sampling was to know their literacy competency level about the Internet on entrance level into university life.

**Fig 2:**
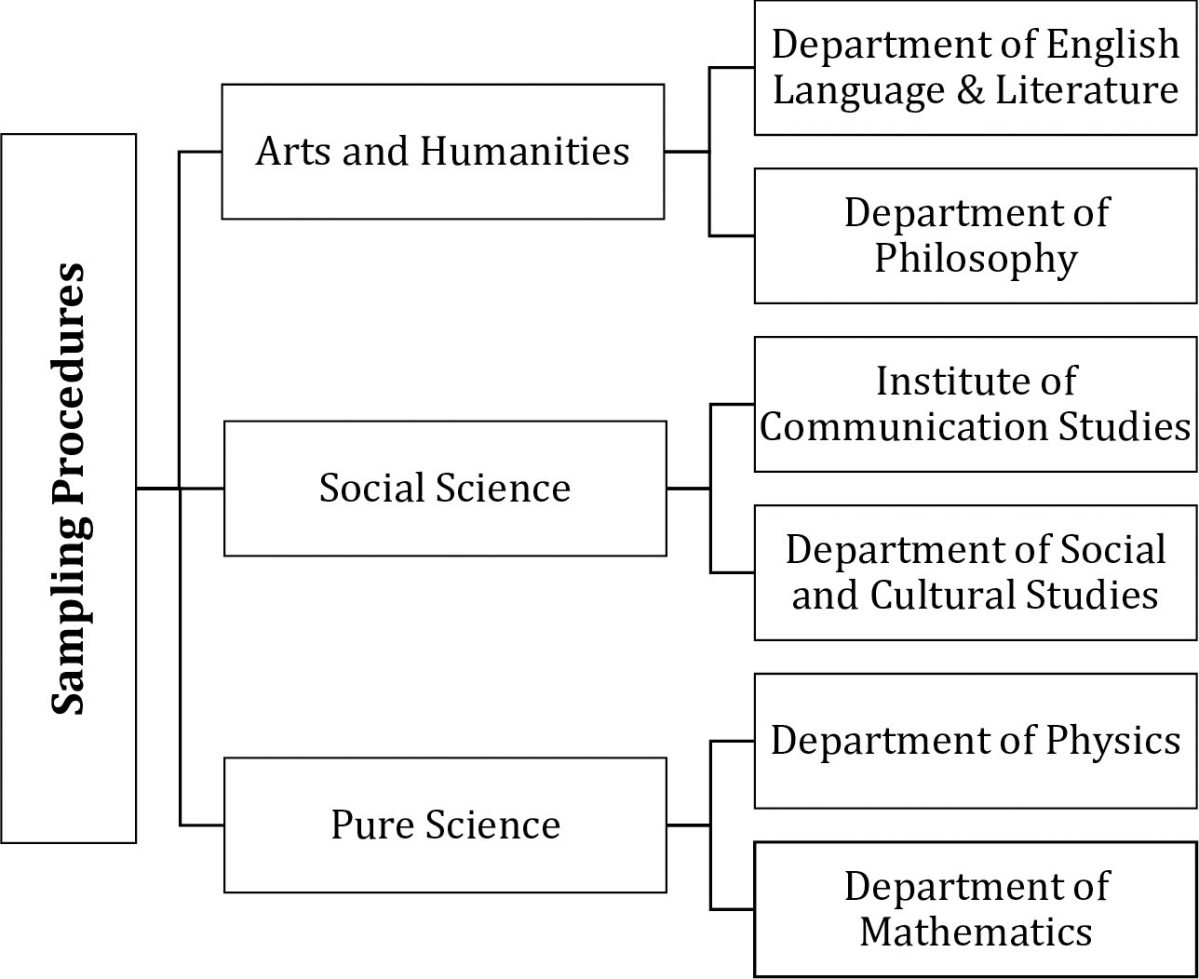
A flow chart of population and sampling procedures.

A total of 2500 students were enrolled in undergraduate programs of the University of the Punjab in 2018. A two-stage sampling strategy was carried out to select a sample (see Fig 2). In the first stage, the knowledge is divided into three disciplines i.e. Pure Sciences, Social Sciences, and Humanities & Arts, and the population was divided into 3 strata, which consisted of 3 main disciplines i.e. Pure Sciences, Social Sciences, and Humanities & Arts. In the second stage, two departments from each discipline were selected on convenience-based. Data were collected from 30 available students (both male and female) of BS first year from each selected department. The total number of respondents was 180.

A questionnaire was developed based on a related literature review and according to the definition of internet literacy. According to the Ministry of Internal Affairs and Communications Japan (2013), Internet Literacy is “the ability necessary to access the Internet while incorporating the following three points: (1) the ability to address illegal and harmful contents on the Internet appropriately, (2) the ability to communicate on the Internet appropriately, and (3) the ability to protect their privacy and perform security measures”.

Since the researchers could not find a questionnaire in the existing literature fulfilling the objectives of the current study, therefore the questionnaire was developed based on available literature on the topic within the domain of the research objectives. The questionnaire after development was sent to three experts for content validity, two experts were from the field of information management, whereas one expert belonged to statistics. A few minor suggestions were received from experts in Information Management. Incorporating experts’ suggestions, the final questionnaire was prepared and sent for pilot testing.

For ensuring ethical considerations, the study was reviewed and approved by the Institute of Information Management, University of the Punjab, Lahore. Informed consent was obtained from all the respondents of the study. Furthermore, the respondents were ensured that their identity will not be disclosed, and the data will be used for research purposes only. Data were collected after taking permission from the concerned departments and after the consent of the respondents. A cover letter for the questionnaire explaining research objectives and fair use of the data was prepared for informed consent, and no personal data of the participants (name, email, phone number) was collected. After reading the cover letter the participants who agreed filled the questionnaires. Pilot testing was done from the Department of Mathematics (30 respondents), University of the Punjab, Lahore which was not part of the research sample, and no problems and errors were reported by the respondents with the test mechanics. No changes were made to the questionnaire after pilot testing. To test the reliability of the constructs Cronbach’s alpha was calculated. Table 1 indicates that Cronbach’s alpha of the constructs remained between .68 to .86 which is considered reliable.

**Table 1:** Reliability of constructs.

Constructs	Alpha
Ability to address illegal and harmful content	0.72
Communication Channels Exposure	0.86
Communication Proficiency	0.76
Data Privacy Settings Awareness	0.68
Data Privacy and Security Behavior	0.69

Final data were collected by one of the researchers personally. The researcher visited the departments and with their permission distributed questionnaires in the BS 1^st^ semester class. Normally, 40 to 50 students are enrolled in a BS 1^st^-semester class. The data were collected from at least 30 available and agreed BS 1^st^ semester students. Thus, 180 students from six departments were recruited. After collection, data were arranged, examined, and construed using SPSS (22.0) to grasp the conclusions. For analysis and interpretation of the data, descriptive and inferential statistics were employed.

## Results

Findings of the study depict that male 94 (52.2%) and female respondents (47.8%) were almost equal in number, and almost all students were daily internet users (94.4%)). Further Results are presented according to the research objectives.

The *first objective* of the study was to ascertain students’ ability to address the illegal and harmful contents on the internet appropriately. To meet this research objective two constructs were measured i.e. Students’ knowledge level about the legal status of online activities and their ability to respond to the illegal and harmful contents.

### Respondents’ knowledge level

To ascertain students’ knowledge level, a series of 20 questions were included about activities on and about the Internet (Appendix A). Respondents had to determine the legal status of internet-based information and activities. Those statements which were answered right according to their legal and illegal value were assigned one mark and which were answered wrong were assigned zero. Knowledge level was ranked into four levels i.e. poor (Less than 50% correct answers), average (51–65% correct answers), good (66–80% correct answers), and very good (Above 80% correct answers). Results in Table 2 indicate that a majority of the students have good knowledge about the legal and illegal status of information and its related Internet activities.

**Table 2:** Knowledge level (n = 180).

Marks	Knowledge to determine the legality of contents	Frequency	Percent
Less than 50%	Poor	26	14.4
51–65%	Average	25	13.9
66–80%	Good	66	36.7
Above 80%	Very Good	63	35.0
Total		180	100

### Respondents’ ability to address illegal and harmful content

Table 3 indicates students’ ability to identify contents prohibited by the law and are capable of causing harm. Results give evidence that students were often able to stay safe (Mean = 3.89, SD = 1.11) and to judge the legal and illegal Internet contents (Mean = 3.84, SD = 1.06) but it is also evident that respondents rarely use filtering software to prevent illegal and harmful Internet contents (Mean = 2.91, SD = 1.53). Data in Table 3 also show that students only sometimes evaluated the validity and authenticity of information (Mean = 3.39, SD = 1.31) and to identify fake websites on the Internet (Mean = 3.16, SD = 1.31). The overall results indicate that respondents possess an average level of ability to stay safe on the Internet and to judge legal, illegal, and harmful content available online.

**Table 3:** Measurement items for the six variables of the internet literacy model.

Variables	Statements	Scale	M(SD)
**Ability to Address Illegal and Harmful Contents**	*I am able to stay safe online*	1 = Never, 2 = Rarely, 3 = Sometimes, 4 = Often, 5 = Always	3.89(1.11)
	*I am able to differentiate between legal and illegal contents*	1 = Never, 2 = Rarely, 3 = Sometimes, 4 = Often, 5 = Always	3.84(1.06)
	*I am able to evaluate the authenticity of information available on the Internet*	1 = Never, 2 = Rarely, 3 = Sometimes, 4 = Often, 5 = Always	3.39(1.31)
	*I am able to identify bogus websites on the Internet*	1 = Never, 2 = Rarely, 3 = Sometimes, 4 = Often, 5 = Always	3.16(1.31)
	*I use filtering software for illegal and harmful contents on the Internet*	1 = Never, 2 = Rarely, 3 = Sometimes, 4 = Often, 5 = Always	2.91(1.53)
**Communication Channels Exposure**	*WhatsApp*	1 = Never, 2 = Rarely, 3 = Sometimes, 4 = Often, 5 = Always	4.59(.97)
	*Google*	1 = Never, 2 = Rarely, 3 = Sometimes, 4 = Often, 5 = Always	4.56(.95)
	*YouTube*	1 = Never, 2 = Rarely, 3 = Sometimes, 4 = Often, 5 = Always	4.34(.96)
	*Facebook*	1 = Never, 2 = Rarely, 3 = Sometimes, 4 = Often, 5 = Always	3.81(1.37)
	*Email*	1 = Never, 2 = Rarely, 3 = Sometimes, 4 = Often, 5 = Always	3.77(1.35)
	*Instagram*	1 = Never, 2 = Rarely, 3 = Sometimes, 4 = Often, 5 = Always	3.18(1.64)
	*Snapchat*	1 = Never, 2 = Rarely, 3 = Sometimes, 4 = Often, 5 = Always	2.59(1.72)
	*Yahoo*	1 = Never, 2 = Rarely, 3 = Sometimes, 4 = Often, 5 = Always	2.49(1.39)
	*Twitter*	1 = Never, 2 = Rarely, 3 = Sometimes, 4 = Often, 5 = Always	2.41(1.47)
	*Skype*	1 = Never, 2 = Rarely, 3 = Sometimes, 4 = Often, 5 = Always	2.34(1.41)
	*Blogs*	1 = Never, 2 = Rarely, 3 = Sometimes, 4 = Often, 5 = Always	2.16(1.42)
**Communication Proficiency**	*Social networking sites (Facebook*, *Twitter*, *Instagram)*	1 = Novice, 2 = Beginner, 3 = Competent, 4 = Proficient, 5 = Expert	2.76(1.30)
	*Media hosting sites (YouTube)*	1 = Novice, 2 = Beginner, 3 = Competent, 4 = Proficient, 5 = Expert	2.39(1.36)
	*Wikis (Wikipedia*, *Wikia)*	1 = Novice, 2 = Beginner, 3 = Competent, 4 = Proficient, 5 = Expert	2.01(1.12)
	*Blogs (Propakistani*, *Techmaish)*	1 = Novice, 2 = Beginner, 3 = Competent, 4 = Proficient, 5 = Expert	2.01(1.31)
	*Products/services review (Apps reviews*, *Book reviews)*	1 = Novice, 2 = Beginner, 3 = Competent, 4 = Proficient, 5 = Expert	1.93(1.12)
**Data Privacy Settings Awareness**	*I know what privacy settings are*	1 = Not at all, 2 = To small extent, 3 = To some extent, 4 = To a moderate extent, 5 = To a great extent	4.06(1.07)
	*I have changed privacy settings so only specified persons can see my profile*	1 = Not at all, 2 = To small extent, 3 = To some extent, 4 = To a moderate extent, 5 = To a great extent	4.04(1.25)
	*I know how to change privacy settings*	1 = Not at all, 2 = To small extent, 3 = To some extent, 4 = To a moderate extent, 5 = To a great extent	3.98(1.06)
	*I take someone’s help to change privacy setting	1 = Not at all, 2 = To small extent, 3 = To some extent, 4 = To a moderate extent, 5 = To a great extent	2.65(1.42)
**Data Privacy and Security Behavior**	*I use secure websites to perform sensitive transactions on public Wi-Fi*	1 = Never, 2 = Rarely, 3 = Sometimes, 4 = Often, 5 = Always	3.65(1.53)
	*I use two-steps verification process for signing in*	1 = Never, 2 = Rarely, 3 = Sometimes, 4 = Often, 5 = Always	3.39(1.43)
	*I read license agreement and privacy terms while registration online*	1 = Never, 2 = Rarely, 3 = Sometimes, 4 = Often, 5 = Always	2.94(1.49)
	*I forget to sign out accounts*	1 = Never, 2 = Rarely, 3 = Sometimes, 4 = Often, 5 = Always	2.76(1.45)
	*I save passwords in my browser	1 = Never, 2 = Rarely, 3 = Sometimes, 4 = Often, 5 = Always	2.67(1.59)
	*I download files from unsecured websites	1 = Never, 2 = Rarely, 3 = Sometimes, 4 = Often, 5 = Always	2.46(1.33)
	*I open emails from people whom I don’t know	1 = Never, 2 = Rarely, 3 = Sometimes, 4 = Often, 5 = Always	2.25(1.39)

*Negative statements were reversed.

The *second research objective* focused on examining the students’ ability to communicate on the Internet appropriately. In this regard, students’ exposure to communication channels and their perceived communication proficiency was investigated.

### Communication channels exposure

Results in Table 3 reveal that WhatsApp (Mean = 4.59, SD = 0.97) and Google are more popular communication mediums among students, alongwith YouTube (Mean = 4.34, SD = 0.96), Facebook (Mean = 3.81, SD = 1.37) and Emails (Mean = 3.77, SD = 1.35). The results further unveil that Instagram (Mean = 3.18, SD = 1.64) and Snapchat (Mean = 2.59, SD = 1.72) are less popular among Pakistani students for communication. Similarly, Yahoo (Mean = 2.49, SD = 1.39), Twitter (Mean = 2.41, SD = 1.47), Skype (Mean = 2.34, SD = 1.41), Linked Inn (Mean = 2.24, SD = 3.91) and Blogs (Mean = 2.16, SD = 1.42) were uncommon medium of communication among Pakistani students.

### Communication proficiency

Data in Table 3 indicate the competency level of the students in terms of content creating or posting through the Internet. Findings show that students were at a competent level to create or post online for social networking sites like Facebook, Twitter, Instagram, etc. (Mean = 2.76, SD = 1.30). However, they are beginners for media hosting sites like YouTube (Mean = 2.39, SD = 1.36), Wikis (Mean = 2.01, SD = 1.12), Blogs (Mean = 2.01, SD = 1.31), and Products or services review (Mean = 1.93, SD = 1.12).

*The third research objective* lead towards the assessment of students’ ability to protect their privacy and perform security measures. Again, two constructs were measured to meet the third research objective of the study i.e. Students’ data privacy settings awareness and their data privacy and security behavior/practices.

### Data privacy settings awareness

Table 3 further illustrates the self-perceived level of students’ knowledge about privacy settings for social networking websites like Facebook, Twitter, Imo, Instagram, WhatsApp, etc. The results in Table 3 indicate that students possess a moderate level of knowledge about the privacy settings (Mean = 4.06, SD = 1.07), customization of privacy settings (Mean = 4.04, SD = 1.25), and in changing privacy settings (Mean = 3.98, SD = 1.06). Further analysis indicates that up to some extent they take help from other people to alter their privacy options (Mean = 2.65, SD = 1.42).

### Data privacy and security behavior

Data in Table 3 uncovers the fact that students often use protected websites to make sensitive transactions on the freely available Wi-Fi facilities (Mean = 3.65, SD = 1.53). The analysis further describes that Pakistani undergraduate students sometimes use a two-step security-authentication process to sign in into different accounts (Mean = 3.39, SD = 1.43) and also forget to sign out from their accounts (Mean = 2.76, SD = 1.45). Results also indicate that students sometimes save accounts’ passwords in different browsers while accessing the Internet (Mean = 2.67, SD = 1.59). However, they rarely download files from unsafe websites (Mean = 2.46, SD = 1.33) and rarely open emails from unknown senders and strange people (Mean = 2.25, SD = 1.39). Results conclude that students sometimes perform activities to protect data and privacy online but do not take privacy measures carefully.

The purpose of the *fourth research objective* was to map a model predicting antecedents of Internet literacy based on the data calculated to fulfill the first three research objectives. For this purpose, a set of six hypotheses was developed for statistical testing.

### Hypotheses testing

A simple linear regression analysis was applied to test each research hypothesis. The results in Table 4 confirm H1, H3, H4, H5, and H6 at p < .05, However, H2 is rejected at p>.05. It is proved that students who use the Internet more frequently possess better levels of knowledge to determine the legal status of the online contents (β = .15, t = 2.10, p < 0.05). However, the knowledge about the legality of online content has no significant impact on students’ ability to address illegal and harmful contents on the Internet (β = .06, t = .92, p > 0.05).

**Table 4:** Hypotheses testing.

	Hypotheses	R^2^	β	t	p	Remarks
H1	Internet Use Frequency -> Knowledge to determine the legality of contents	.024	.15	2.10	.037*	**Accepted**
H2	Knowledge to determine the legality of contents -> Ability to Address Illegal and Harmful Contents on the Internet	.005	.06	.92	.356	**Rejected**
H3	Internet Use Frequency -> Communication Channels Exposure	.040	.19	2.70	.007*	**Accepted**
H4	Communication Channels Exposure -> Communication Proficiency	.048	.16	2.29	.023*	**Accepted**
H5	Internet Use Frequency -> Data Privacy Settings Awareness	.038	.19	2.66	.008*	**Accepted**
H6	Data Privacy Settings Awareness -> Data Privacy Behavior	.090	.30	4.19	.000*	**Accepted**

Note: *p < .05.

The results in Table 4 further confirm that more use of the Internet leads to more frequent exposure to communication channels (β = .19, t = 2.70, p < 0.05). Likewise, more exposure to communication channels predicts better communication proficiency (β = .16, t = 2.29, p < 0.01).

**Fig 3:**
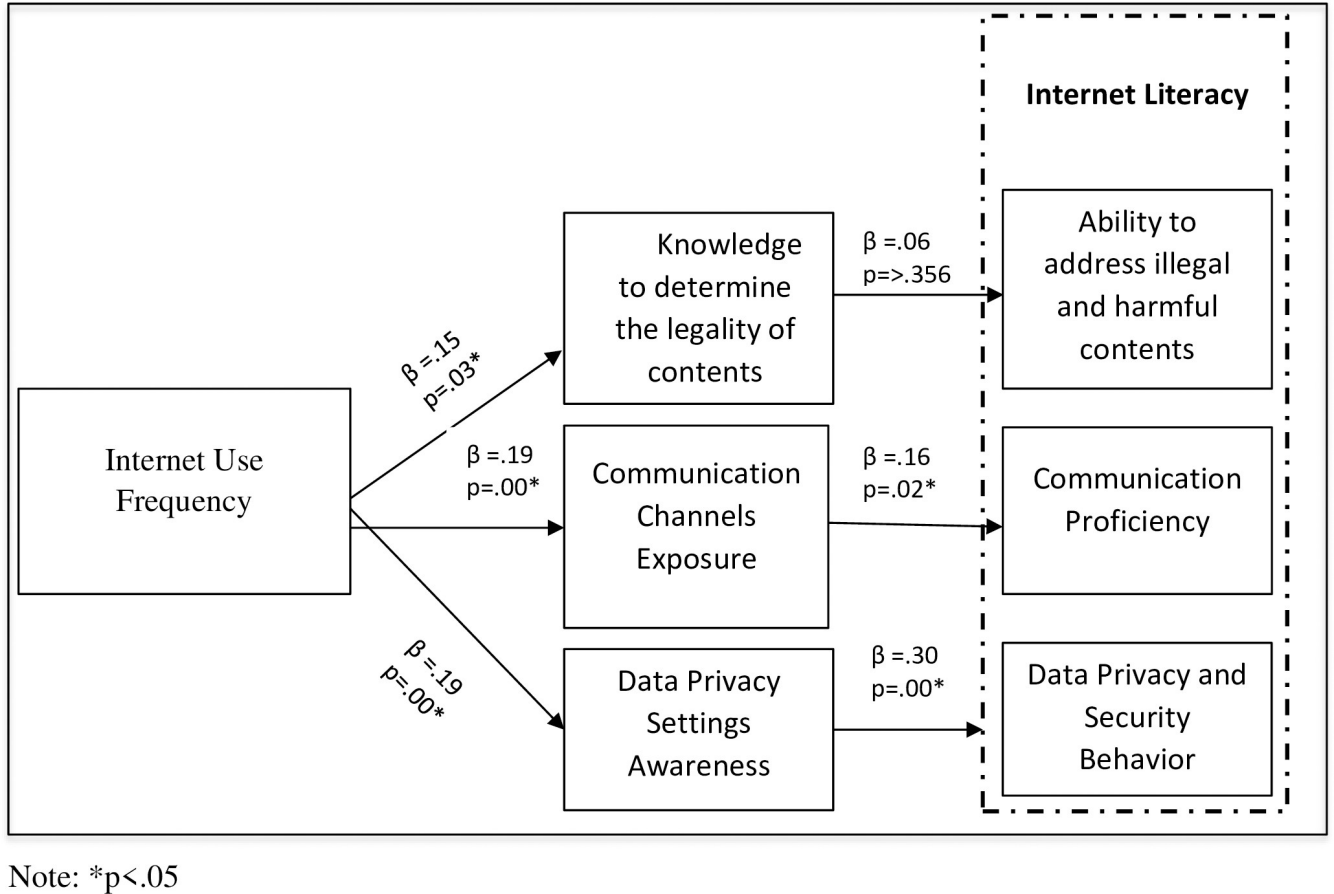
Internet literacy mode.

Frequency of Internet use has also a positive significant impact on knowledge about data privacy (β = .19, t = 2.66, p < 0.05). Knowledge about data privacy impacts the data privacy behaviour of students (β = .30, t = 4.19, p < 0.01). Path analysis is presented in Fig 3.

## Discussion and conclusion

The study aimed to explore the Internet literacy competencies of BS first-year students and to map a model predicting antecedents of Internet Literacy. The results showed that undergraduate students are regular Internet users. Furthermore, in terms of knowledge regarding online legal and illegal contents and activities, digital natives have good knowledge and understanding. They not only know how to stay safe but have the ability to differentiate between legal and illegal content available on the Internet. However, their self-perceived ability to address illegal and harmful contents was not up to a satisfactory level, and the use of content filtering or spyware detecting software was very low. Another study from Pakistan reported that reporting behavior of cybercrime is very low among university students [29]. However, this behavior is not common in Pakistani culture only, as Australian university students were also found not reporting cyberbullying [30]. Similarly, United Arab Emirates’ only 39% of the studied university students mentioned that they prefer to report cyberbullying [31].

Whatsapp, Google, and Youtube are the most frequently exposed channels for communication among students. Whereas, a recent study [32] found Youtube, Facebook, and Instagram as the most accessed social media sites among digital native students. In the Pakistani context, Whatsapp is on the top followed by Youtube and Facebook, however, Instagram remained a less common communication channel among Pakistani digital natives.

Although students believed that they are sufficiently proficient in communicating on social media sites like Facebook and Whatsapp, however, they are beginners in communication on media hosting sites like Youtube, Wikipedia, and Blogs. These media hosting sites are particularly used for academic purposes.

The study further reveals that students’ self-perceived knowledge about privacy settings of the accounts was at a moderate extent. It is much important to note that students’ self-reported activities for data privacy and security were not very secure. This finding is in line with the results of previous studies [33–35] that people often report better awareness levels about social media privacy settings but in practice, they practice less secure activities.

It can be safely concluded that though students have knowledge about illegal/harmful content on Internet, still their ability to address these contents, their communication proficiency, and data security behavior is not up to a satisfactory level. It infers that there is a dire need to address the matter to save digital natives from cybercrimes, cyberbullying, etc., and to help them in effective and efficient use of the Internet for their personal and academic purposes.

The Internet literacy model proposed and tested in the current study confirms “Internet Use Frequency”, “Communication Channels Exposure”, “Knowledge about data security and Privacy” as predictors of “Knowledge about legality of online contents”, “Communication Proficiency” and “Data Security and Privacy Behavior”.

### Research implications

The study offers twofold implications, first theoretical and second practical. Theoretically, the study decomposed the definition of “Internet Literacy” making it clearer and more understandable, coupling the knowledge with the practice of Internet literacy skills. Furthermore, a model has been developed predicting the antecedents of internet literacy. It is a significant contribution in the literature of internet literacy that could not be found in previously published literature.

Practically, the study suggests that knowledge about data privacy and security contributes to data security behavior, therefore, awareness campaigns addressing the potential for illegal and harmful use of the Internet should be started. Internet literacy, as a part of information literacy, may be included in the coursework by departments/institutes/centers/colleges/universities. The integration of modules on Internet safety and Internet Ethics is required into the curriculum of students at school/college levels. Seminars and workshops should be conducted with the help of the cyber-crime investigation department for awareness purposes and to encourage students’ morale and confidence to report illegal material to them. For digital natives, coordinated educational efforts to combine formal educational curriculum and Internet literacy education is much needed to attain opportunities and to realize possibilities in the mediatized world [36].

As exposure to communication channels is correlated with communication proficiency, therefore, the academic institutions should encourage students the academic use of these communication mediums. Facebook, Whatsapp, Blogs, etc. can be used for academic information purposes. Exposure to these sites may help them to develop their Internet communication proficiency under the supervision of their teachers. As the academic institutions are spending a handsome amount on IT infrastructure to facilitate students, the above-mentioned implications will help Universities to achieve the required results more efficiently.

### Future topics

The topics for further researcher are as follows.

To compare the results of entry point students, the study should be conducted on BS final year students of the University of the Punjab, Lahore exploring their Internet literacy skills. The understudy students were from the first year of BS, a comparative study should be done after three years from the same students to identify any difference in their Internet literacy skills. The study should also be conducted on a large scale to explore the Internet literacy skills of the students of different universities, colleges, and institutes, so that the results may be generalized.

### Limitations

This study is conducted on a small sample from the University of the Punjab (PU), Lahore. The results may not be generalized to all undergraduate students (digital natives).

## Appendix A

### Questionnaire

**Table:** Please tick (√) the most appropriate option.

How often do you use internet?	**Daily**	**Weekly**	**Monthly**	**Rarely**	**Never**

### 1. Illegal/Harmful Contents

**(1.1):** What is your opinion about the following information/activities?

Information/activities	Legal/Not Harmful	Illegal/Harmful
Copyright theft		
Blocking/filtering illegal contents		
Financial scams (i.e. Credit card fraud, Money laundering)		
Downloading and using files with permission		
Cyber terrorism		
Pirating (i.e. Circulating illegal copies of software)		
Fight against terrorism and hate speech		
Copyright protection		
Cyberbullying someone via social media		
Searching for required information online		
Online gambling (i.e. Poker, Casinos, Sports betting)		
Drugs related information/activities		
Suicide assistance		
Downloading movies/books for personal use with permission		
Insulting/Offensive contents		
Parody accounts (i.e. fake accounts on Facebook, Twitter)		
Website Hacking		
Accessing streamed contents from streaming sites		
Bomb making instructions		
Using unsecure Wi-Fi networks		

Any other (Please Specify)

**(1.2):** The ability to address illegal and harmful contents on the Internet appropriately.

Statements	Always	Often	Sometimes	Rarely	Never
I am able to stay safe online					
I am able to differentiate between legal and illegal contents					
I am able to identify bogus websites on the internet					
I am able to evaluate the authenticity of information available on the internet					
I use filtering software for illegal and harmful contents on the internet (i.e. antivirus software)					

### 2. Communication Through Internet

**(2.1):** How often do you use following internet channels for communication?

**Internet channels**	**Always**	**Often**	**Sometimes**	**Rarely**	**Never**
Google					
Yahoo					
YouTube					
E-mail					
Skype					
Facebook					
Twitter					
Instagram					
LinkedIn					
Snapchat					
Blogs					
WhatsApp					

Any other (Please Specify)

**(2.3):** How much you are proficient to create or post contents online on?

Forums	Novice	Beginner	Competent	Proficient	Expert
Product/services review (i.e. Apps reviews, Book reviews)					
Wikis (i.e. Wikipedia, Wikia)					
Social networking sites (i.e. Facebook, Twitter, Instagram)					
Media hosting sites (i.e. YouTube)					
Blogs (i.e. Propakistani, Techmaish)					

### 3. Data Privacy and Protection

**(3.1):** Please rate your knowledge about privacy settings for social networking sites.

Statements	To a great extent	To a moderate extent	To some extent	To small extent	Not at all
I know what privacy settings are					
I know how to change privacy settings					
I have changed privacy settings so only specified persons can see my profile					
I take someone’s help to change privacy settings					

**(3.2):** How often do you perform following activities for online data privacy and protection?

Activities	Always	Often	Sometime	Rarely	Never
**I use secure websites to perform sensitive transactions on public Wi-Fi**					
**I forget to sign out accounts**					
**I open emails from people whom I don’t know**					
**I use two-steps verification process for signing in**					
**I download files from unsecure websites**					
**I save passwords in my browser**					
**I read license agreement and privacy terms while registration online**					

### Demographic Information

**Table:** 

Department/Institute/College				
Program				
Sex/gender	Male		Female	
Age (In years)				

**Thanks for your cooperation**.

## Supporting information

S1 File Anonymous data internet literacy. (SAV)
